# CRISPR Interference for Rapid Knockdown of Essential Cell Cycle Genes in *Lactobacillus plantarum*

**DOI:** 10.1128/mSphere.00007-19

**Published:** 2019-03-20

**Authors:** Ine Storaker Myrbråten, Kamilla Wiull, Zhian Salehian, Leiv Sigve Håvarstein, Daniel Straume, Geir Mathiesen, Morten Kjos

**Affiliations:** a Faculty of Chemistry, Biotechnology and Food Science, Norwegian University of Life Sciences, Ås, Norway; University of California, Davis

**Keywords:** CRISPRi, *Lactobacillus plantarum*, *acm2*, bacterial cell cycle, *cozE*, *dnaA*, *eloR*, *ezrA*, *khpA*, knockdown

## Abstract

Studies of essential genes in bacteria are often hampered by the lack of accessible genetic tools. This is also the case for Lactobacillus plantarum, a key species in food and health applications. Here, we develop a clustered regularly interspaced short palindromic repeat interference (CRISPRi) system for knockdown of gene expression in L. plantarum. The two-plasmid CRISPRi system, in which a nuclease-inactivated Cas9 (dCas9) and a gene-specific single guide RNA (sgRNA) are expressed on separate plasmids, allows efficient knockdown of expression of any gene of interest. We utilized the CRISPRi system to gain initial insights into the functions of key cell cycle genes in L. plantarum. As a proof of concept, we investigated the phenotypes resulting from knockdowns of the cell wall hydrolase-encoding *acm2* gene and of the DNA replication initiator gene *dnaA* and of *ezrA*, which encodes an early cell division protein. Furthermore, we studied the phenotypes of three cell division genes which have recently been functionally characterized in ovococcal bacteria but whose functions have not yet been investigated in rod-shaped bacteria. We show that the transmembrane CozE proteins do not seem to play any major role in cell division in L. plantarum. On the other hand, RNA-binding proteins KhpA and EloR are critical for proper cell elongation in this bacterium.

**IMPORTANCE** L. plantarum is an important bacterium for applications in food and health. Deep insights into the biology and physiology of this species are therefore necessary for further strain optimization and exploitation; however, the functions of essential genes in the bacterium are mainly unknown due to the lack of accessible genetic tools. The CRISPRi system developed here is ideal to quickly screen for phenotypes of both essential and nonessential genes. Our initial insights into the function of some key cell cycle genes represent the first step toward understanding the cell cycle in this bacterium.

## Abstract (precis)

L. plantarum is an important bacterium for applications in food and health. Deep insights into the biology and physiology of this species are therefore necessary for further strain optimization and exploitation; however, the functions of essential genes in the bacterium are mainly unknown due to the lack of accessible genetic tools. The CRISPRi system developed here is ideal to quickly screen for phenotypes of both essential and nonessential genes. Our initial insights into the function of some key cell cycle genes represent the first step toward understanding the cell cycle in this bacterium.

## INTRODUCTION

The lactic acid bacterium (LAB) Lactobacillus plantarum is an important species for food and health applications. L. plantarum is naturally found in a variety of habitats, including meat and dairy products and fermented vegetables and in the oral cavity and gastrointestinal tract of humans (1–3). It has been documented that L. plantarum strains have probiotic effects on humans (4–6), and at least some strains have been shown to modulate the immune system (7). Furthermore, extensive research has been performed in recent decades in investigations of LAB, including L. plantarum, as live delivery vehicles for therapeutic molecules such as antigens, cytokines, and antibodies (8–11). Given the importance and the potential new applications of L. plantarum, there is a need to develop strains with improved growth, robustness, and protein secretion capacities.

Such strain development heavily relies on further insights into the biology of these cells. Most studies on L. plantarum have been performed in the model strain WCFS1 (12), which was the first *Lactobacillus* strain whose genome was sequenced. This strain is easily transformable by electroporation, and tools for plasmid-based expression platforms are available, including inducible expression systems based on bacteriocin regulatory systems (pSIP, pNICE) (13–16). The high transformation efficiency has also allowed the construction of a number of isogenic mutants in genes involved in different pathways and functions. In particular, the Cre-*lox* system, which is based on double-crossover gene replacement, has been important in this field (17), although mutants have also been made using suicide vectors (18). Mutant construction in L. plantarum is, however, a laborious and time-consuming process, and novel methods for phenotyping are highly desirable.

**FIG 1:**
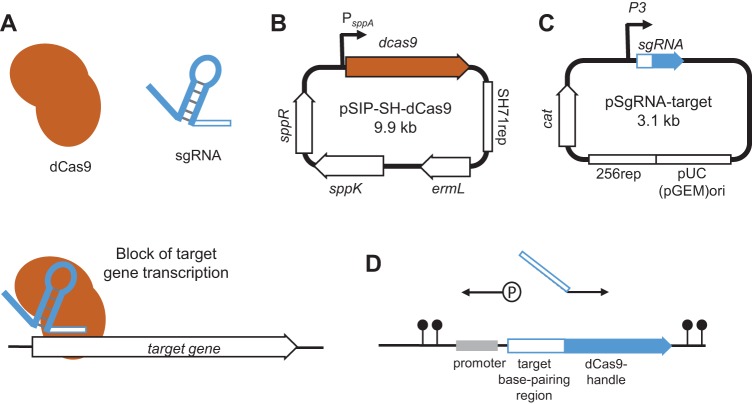
The two-plasmid CRISPRi-system. (A) Schematic presentation of transcriptional knockdown by CRISPRi. Block of RNA polymerase and transcription occurs when dCas9 (orange) and the sgRNA (blue) bind specific sites in the 5′ end of the target gene, guided by the 20-nucleotide (nt) sgRNA sequence. (B) Overview of pSIP-SH-dCas9 plasmid. The *dcas9* gene is located downstream of the inducible *sppA* promoter (P*_SppA_*). The two-component regulatory genes, *sppK* and *sppR*, are located on the same plasmid. “*ermL*” and “SH71_rep_” indicate the erythromycin resistance gene and the replicon determinant, respectively. (C) Overview of the prototype expression vector of sgRNA. The sgRNA is constitutively expressed from promoter P3. “*cat*” and “256_rep_/pUC(pGEM)ori” indicate the chloramphenicol resistance gene and the replicon determinant, respectively. Both plasmids (see panels B and C) were transformed into L. plantarum to achieve transcriptional knockdown of the target gene. (D) A detailed view of the sgRNA-region in pSgRNA-target. The gene-specific target region (white) and dCas9-handle region (blue) of the sgRNA are shown downstream of the cognate promoter (gray). Terminator sequences are indicated by lollipops. New sgRNA plasmids were constructed by inverse-PCR using two primers as indicated by arrows in the figure, with one phosphorylated (P) reverse primer annealing immediately upstream of the targeting-region and one nonphosphorylated forward primer annealing to the dCas9-handle region, containing a 20-nt overhang which is specific to a target gene.

Here we have developed a gene knockdown method known as clustered regularly interspaced short palindromic repeat interference (CRISPRi) in L. plantarum WCFS1 that permits easy downregulation of any gene of interest (19, 20), and, most importantly, it allows studies of essential genes. CRISPRi exploits the CRISPR/Cas9 system by utilizing a catalytically inactive Cas9 protein (dCas9) together with a single guide RNA (sgRNA) that harbors an easily replaceable 20-nucleotide (nt) base-pairing region and a Cas9-handle region. The 20-nt base-pairing region is selected to target the gene of interest, and the sgRNA can easily be redesigned to target any gene of interest. The dCas9 will have lost its ability to cleave DNA, but the DNA-binding property of this protein remains intact. Expression of *dcas9* together with sgRNA thus causes a transcriptional blocking of the RNA polymerase, leading to knockdown of gene expression of the target gene (19, 20) (Fig. 1A). CRISPRi has been successfully established in bacterial species such as Escherichia coli (20), Bacillus subtilis (21), Streptococcus pneumoniae (22), Staphylococcus aureus (23–26), and Lactococcus lactis (27). Note that CRISPR-based tools have been used in lactobacilli previously. In Lactobacillus casei, a nickase Cas9 was used for genome editing (28), while in L. plantarum, genome editing was performed by recombineering double-stranded DNA templates into target sites using cleavage by Cas9 (29). Notably, the latter work demonstrated that the outcomes of the CRISPR experiments could vary between different L. plantarum strains.

While L. plantarum has been extensively studied with respect to host cell interaction, immune cell modulation, protein secretion, biofilm formation, interaction with food components, and production of bacteriocins (30), much less is known about essential processes of the bacterial cell cycle in these rod-shaped bacteria. Most of our knowledge on the cell cycle of Gram-positive, rod-shaped bacteria comes from B. subtilis, where DNA replication, chromosome segregation, cell wall synthesis, and cell division, as well as the coordination of these processes, have been investigated in detail (31–33). Although L. plantarum is related to B. subtilis, there are also key differences between the cell cycles of these species, for example, with regard to sporulation. Specific knowledge about the functions of proteins and factors affecting cell cycle processes in L. plantarum is therefore important, since such knowledge may pave the way for development of strains with improvements with respect to protein secretion or interactions with host cells (18).

In this study, we utilized the CRISPRi system to get initial insights into the functions of putative cell cycle proteins in L. plantarum. As a proof of principle, we studied proteins hypothesized to affect different stages of the bacterial cell cycle. These included (i) Acm2, a cell wall hydrolase previously shown to play a major role in daughter cell separation in L. plantarum (34, 35); (ii) the bacterial DNA replication initiator protein DnaA (36); and (iii) the early cell division protein EzrA. EzrA is a membrane-associated protein involved in coordination of cell division and cell wall synthesis in Gram-positive bacteria (37). The CRISPRi system was also used to study the functions of proteins putatively involved in bacterial cell elongation but whose functions have not previously been studied in rod-shaped bacteria. These proteins, named CozE (38), EloR (39), and KhpA (40), have all been identified as essential for proper cell elongation in the oval-shaped bacterium S. pneumoniae. CozE (coordinator of zonal elongation) has been shown to control cell elongation by directing the activity of cell wall synthesizing proteins (38, 41). Homologs of CozE (CozEa and CozEb) are also essential for proper cell division in S. aureus (23). EloR (elongation regulator) and KhpA (KH-containing protein A) are two cytoplasmic, RNA-binding proteins which form a midcell-localized heterocomplex (39, 40, 42).

## RESULTS AND DISCUSSION

### Construction of a two-plasmid system for CRISPR interference in L. plantarum.

Given the lack of tools for fast and easy depletion of genes in L. plantarum, we developed a two-plasmid CRISPRi system for this purpose (Fig. 1). The system is based on the CRISPRi systems developed for S. pneumoniae (22) and S. aureus (23). This CRISPR/Cas9-based system can be utilized in L. plantarum WCFS1, since this strain does not encode any native CRISPR/Cas9 (12). For expression of nuclease-inactive Cas9 (dCas), *dcas9* was cloned under the control of the inducible promoter *sppA* in the plasmid pSIP403 (15), harboring an erythromycin resistance gene, *ermL*, as well as *sppK* and *sppR*, encoding a histidine protein kinase and a response regulator, respectively. By external addition of the inducer peptide SppIP, expression from promoter *sppA* is induced via this two-component regulatory system (15). The replicon 256_rep_ in pSIP403 was exchanged with the lactococcal SH71_rep_ replicon (Fig. 1B) to make it compatible with the sgRNA-target plasmid, which also is a derivative of pSIP403.

The sgRNA plasmids contain a chloramphenicol resistance gene (*cat*) and an sgRNA cassette under the control of a synthetic, constitutively expressed promoter, P3 (22) (Fig. 1C). The sgRNA cassette includes a 20-nt base-pairing region and a dCas9-handle region (Fig. 1D). The sgRNA-target sequence can easily be exchanged using inverse-PCR with a forward primer containing the gene-specific 20-nt base-pairing region as an overhang (Fig. 1D). The sgRNA has the capability of binding target DNA by complementary base pairing and dCas9 through the secondary structure of the Cas9-handle. Induced expression of dCas9 leads to formation of a sgRNA-dCas9-DNA complex that acts as a physical blockage for RNA polymerase (Fig. 1A). In all cases, the sgRNAs were designed to target a location close to the 5′ end of the gene of interest. The replicon of the sgRNA plasmids (256_rep_) has a narrow host range which has been shown to work in only a few *Lactobacillus* species, namely, L. plantarum, Lactobacillus sakei, and Lactobacillus curvatus (43).

**FIG 2:**
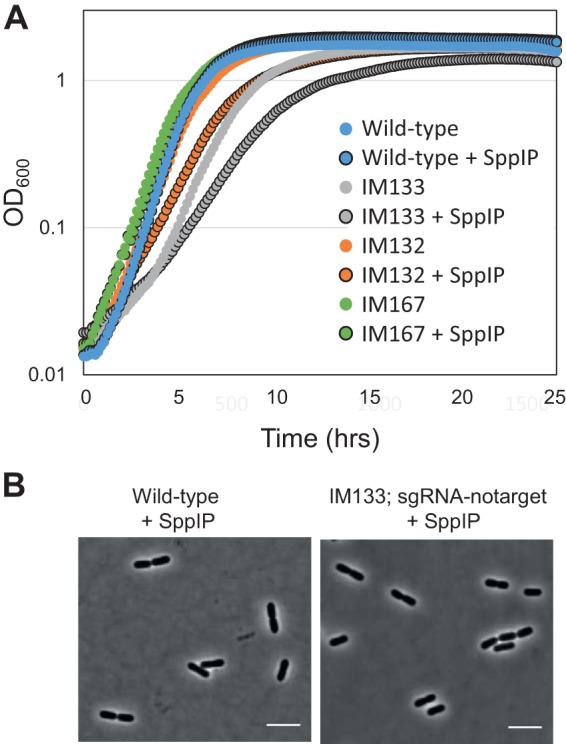
Analysis of dCas9 expression. (A) Growth analysis of wild-type L. plantarum WCFS1 (blue), IM133 (WCFS1 carrying pSIP-SH-dCas9 and pSgRNA-notarget; gray), IM132 (WCFS1 carrying only pSIP-SH-dCas9; orange), and IM167 (WCFS1 carrying pEV and pSgRNA-notarget; green). Levels of growth of noninduced cultures and induced cultures (25 ng/ml inducer pheromone SppIP; black outline) are shown. (B) Phase-contrast micrographs of wild-type L. plantarum WCFS1 cells and IM133. The scale bars represent 5 µm.

High production levels of heterologous proteins using the pSIP expression system have been shown previously to cause growth retardation in L. plantarum WCFS1 (13). Expression of the CRISPRi system with a nontargeting sgRNA (IM133; pSIP-SH-dCas9 and pSgRNA-notarget) indeed resulted in growth reduction (Fig. 2A). In fact, induction of dCas9 expression alone (IM132; pSIP-SH-dCas9) caused a growth defect, while the combination of an empty pSIP vector (pEV) with a nontargeting sgRNA plasmid (IM167; pEV and pSgRNA-notarget) did not. This clearly indicates that overproduction of dCas9 results in impaired growth of L. plantarum. The morphologies of IM133 cells were not affected compared to those of the wild-type cells (Fig. 2B, see below); however, we cannot exclude the possibility that other cellular processes are affected upon dCas9 production in these cells.

### Characterizing the CRISPRi system by targeting *acm2*.

We tested the CRISPRi system by targeting a cell division gene with a known knockout phenotype. Previous work had shown that deletion of the gene coding for the major autolysin Acm2 (*lp_2645*), a cell wall hydrolase (N-acetylglucosaminidase) important for daughter cell separation, results in cell chaining and sedimentation of cultures (34, 44). We constructed a strain targeting *acm2* (IM134, pSIP-SH-dCas9 and pSgRNA-acm2) and analyzed the knockdown effects using the wild-type strain and a nontargeting CRISPRi strain as controls (IM133; pSIP-SH-dCas9 and pSgRNA-notarget).

**FIG 3:**
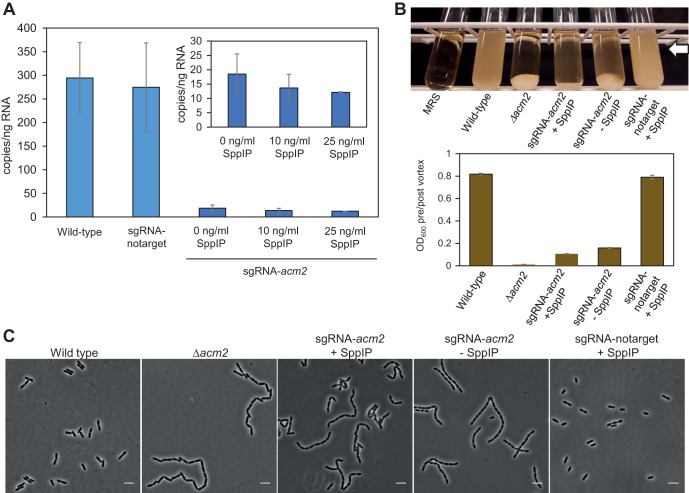
Using CRISPRi to knock down *acm2* expression in L. plantarum WCFS1. (A) Quantifying the expression of *acm2* using droplet digital PCR. The transcriptional levels of *acm2* in wild-type L. plantarum WCFS1, in control cells with nontargeting sgRNA (IM133), and in CRISPRi strain cells with sgRNA targeting *acm2* (IM134) are indicated. For the latter, cultures with SppIP inducer concentrations of 10 and 25 ng/ml were compared to uninduced culture, and those results are also shown (inset). The error bars represent standard deviations of results from at least two biological replicates, each of which was analyzed with three technical replicates. (B) Growth of L. plantarum with various expression levels of *acm2*. (Top panel) Culture tubes of L. plantarum grown for 16 h. Sedimentation of cells was clearly observed when *acm2* was deleted (Δ*acm2* [34]) or knocked down (IM134 with or without added SppIP) but not in the wild-type or control cells (IM133). (Bottom panel) Quantification of cell sedimentation by measuring OD_600_ in the upper layer of the medium (see arrow in top panel) before and after vortex mixing. The ratios are plotted in the bar plot. The error bars represent standard deviations of data from three parallel measurements. (C) Phase-contrast images of the corresponding strains. The CRISPRi strains were imaged at the exponential-growth phase. The scale bar is 5 µm.

First, to test the efficiency of the transcriptional knockdown, we performed droplet digital PCR (ddPCR). Knockdown of *acm2* was achieved in strain IM134, and the results were compared to the control strains (Fig. 3A). Notably, the knockdown was highly effective even without induction, showing that leakage from the *sppA* promoter was sufficient to efficiently drive the CRISPRi system. Only a slight additional decrease in transcription was observed upon induction (10 or 25 ng/ml SppIP) (Fig. 3A, inset).

We next monitored cell sedimentation by growing strains in standing cultures at 37°C for 16 h (Fig. 3B). To quantify the sedimentation, the optical density at 600 nm (OD_600_) was measured in the upper part of the culture volume before and after vortex mixing. In a homogenous culture, the ratio should be close to 1, which was the case for the wild-type WCFS1 and IM133 control cells. In contrast, deletion strain Δ*acm2* showed full sedimentation of cells and a ratio close to zero. Similarly to the results seen with mutant Δ*acm2*, depletion of *acm2* in strain IM134 exhibited a high degree of sedimentation with a ratio at 0.1, reflecting that this is a knockdown and not a knockout of *acm2*. Notably, the noninduced cultures and the induced cultures of IM134 displayed similar sedimentation phenotypes. Furthermore, microscopy analyses of the same strains revealed that knockdown of *acm2* by CRISPRi resulted in a chaining phenotype similar to that seen with knockout mutant Δ*acm2*; since daughter cell separation was inhibited, the cells formed long chains compared to the control (Fig. 3C). Also here, similar phenotypes were observed in induced and noninduced cultures.

Together, these results show that the CRISPRi system functions efficiently and that low basal expression from the *sppA* promoter (16, 45) is sufficient for transcriptional knockdown in L. plantarum WCFS1. Only a small additional knockdown effect was observed after external addition of inducer SppIP, which indicates that the basal promoter activity resulted in production of sufficient numbers of dCas9 proteins for efficient knockdown.

### Knockdown of *dnaA* and *ezrA*.

The results described above show that the CRISPRi system is suitable for transcriptional knockdown of genes in L. plantarum and that the system can be used to study phenotypes of cells when a key cell division gene is depleted. However, *acm2* is a nonessential gene in L. plantarum, since a deletion mutant could be constructed (34). In addition to its easy introduction into L. plantarum, the main strength of a CRISPRi knockdown system is that it allows studies of phenotypes of essential genes in this bacterium. As a further proof of concept, and to gain further insights into the suitability of the CRISPRi system to study essential processes in L. plantarum, we designed sgRNA plasmids to target well-studied bacterial cell cycle proteins, namely, the DNA replication initiator DnaA and the early cell division protein EzrA.

**FIG 4:**
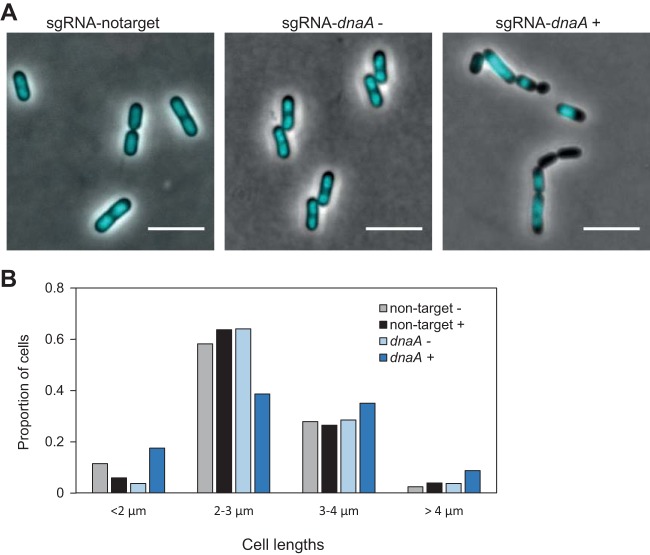
Effect of depleting replication initiation factor *dnaA* in L. plantarum. (A) Micrographs (overlay of phase-contrast and DAPI images) of control cells (strain IM133) and *dnaA*-depleted cells (strain IM137) grown without or with inducer. Cells were grown in the presence of 25 ng/ml SppIP. The scale bar is 5 µm. (B) Cell length analysis of *dnaA* knockdown cells (*n* = 194) compared to control cells (*n* = 204) and noninduced cells (*n* = 320). Cell lengths were measured using MicrobeJ (59). Cell populations were split in four groups based on lengths (<2 µm, 2 to 3 µm, 3 to 4 µm, and >4 µm), and the proportion of cells in each group is plotted. Plus signs (+) and minus signs (−) indicate that cells were grown in the presence and absence of 25 ng/ml SppIP, respectively.

DnaA (*lp_0001*) is essential for initiation of DNA replication from *oriC* in bacteria and has been studied in a number of different bacterial species, including B. subtilis (31). We investigated the CRISPRi knockdown phenotype of *dnaA* by microscopy of cells stained with the nucleoid marker DAPI (4′,6-diamidino-2-phenylindole). As expected, DnaA seems to be essential for proper DNA replication in L. plantarum (Fig. 4A). Upon induction with SppIP, the growth rate was reduced and we observed a large fraction of cells (59.7%, *n* = 139) that were anucleate or displayed abnormal nucleoid morphologies. Such a phenotype was observed neither in the IM133 control cells (0.2% abnormal nucleoids, *n* = 250) nor in the uninduced cells (5.9% abnormal nucleoids, *n* = 320). The latter demonstrates that in the case of *dnaA* knockdown, the induction of dCas9 by SppIP is necessary to observe the full depletion phenotype. Interestingly, the cell morphologies of the DnaA-depleted cells were also severely perturbed. Cell lengths were measured based on the phase-contrast micrographs. This showed that the majority of control cells were between 2 µm and 4 µm in length; however, in the *dnaA* knockdown strain after induction, the fractions of both short (<2-µm) cells and long (>4-µm) cells were larger (Fig. 4B). This supports the notion that proper DNA replication and chromosome segregation are essential for proper cell division, since all cell cycle processes are interlinked (32).

**FIG 5:**
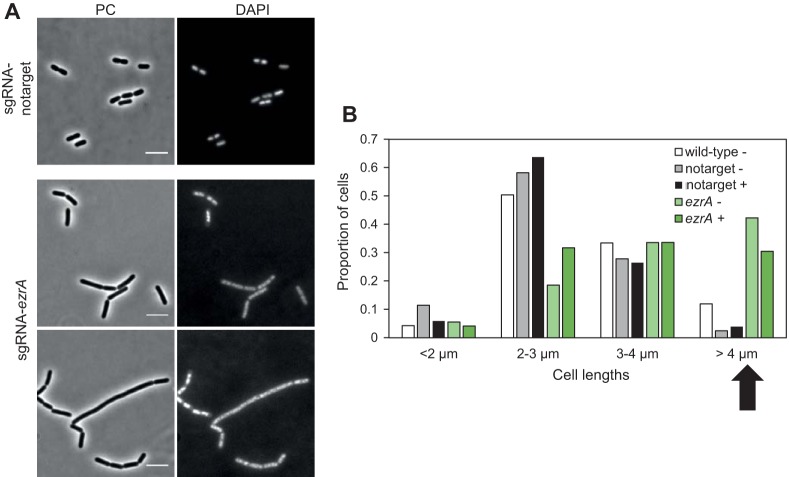
Effect of depleting the cell division protein EzrA in L. plantarum. (A) Phase-contrast (PC) micrographs of cells depleted of *ezrA* (IM188) compared to control cells (IM133). DAPI staining of nucleoids is also shown. The scale bar is 5 µm. (B) Cell length analysis of *ezrA* knockdown cells compared to control cells (IM133) and wild-type cells. Cell lengths were measured using MicrobeJ (59). Cell populations were split in four groups based on lengths, and the proportion of cells in each group is plotted. The black arrow points to the large-cell group (cells = >4 µm), where *ezrA*-depleted cells are clearly overrepresented compared to the control cells. Plus signs (+) and minus signs (−) indicate SppIP-induced and uninduced cells, respectively. Numbers of cells analyzed were as follows: *n* = 377 for wild-type cells, *n* = 122 for notarget control cells (strain IM133) (uninduced), *n* = 204 for notarget control cells (strain IM133) (induced), *n* = 506 for sgRNA-ezrA cells (strain IM188) (uninduced), and *n* = 413 for sgRNA-ezrA cells (strain IM188) (induced).

Next, we made a CRISPRi knockdown strain targeting *ezrA* (*lp_2328*). EzrA is known to interact with a number of different proteins, including FtsZ and penicillin binding proteins, for coordination of cell division and cell wall synthesis in Gram-positive bacteria (46, 47). The growth rate of the *ezrA* knockdown strain was not significantly reduced compared to the control. Cell lengths of the knockdown strain were then analyzed using phase-contrast microscopy. While more than 90% of control cells were between 2 µm and 4 µm in length, up to 40% of the cells were longer than 4 µm in the *ezrA* depletion strain (Fig. 5). The elongated cells were found both with and without induction with SppIP. A similar phenotype was previously reported in B. subtilis (37, 48); B. subtilis cells lacking *ezrA* had delayed cell division and were thus longer than wild-type cells (37, 48). On the other hand, this was not observed in a conditional *ezrA* knockdown in Listeria monocytogenes (49), another rod-shaped Gram-positive species. Together, the data suggest that the role of EzrA in cell division may vary between different rod-shaped bacterial species.

### Depletion of CozE homologs does not perturb division in L. plantarum.

**FIG 6:**
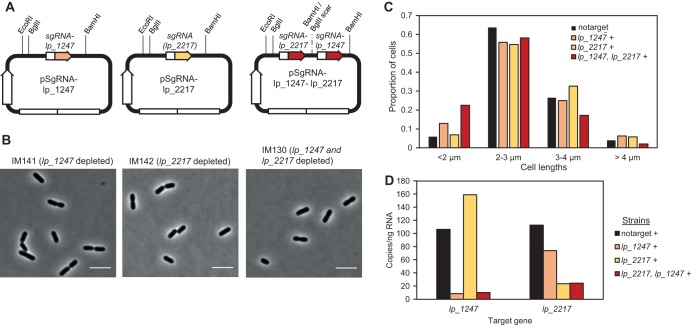
CozE homologs do not dramatically affect cell division in L. plantarum. (A) Schematic overview of plasmids harboring sgRNA to knock down expression of *cozE* homologs *lp_1247* (strain IM141) and *lp_2217* (strain IM142) and double knockdown of *lp_1247* and *lp_2217* (strain IM130). The restriction sites utilized for construction of the double-sgRNA plasmids are indicated (see Materials and Methods for details). (B) Phase-contrast micrographs of cells depleted of *lp_1247* or *lp_2217* or both. Cells were grown in the presence of 25 ng/ml SppIP. The scale bar is 5 µm. (C) Cell length analysis of the corresponding strains compared to control cells (strain IM133). Cell lengths were measured using MicrobeJ (59). Cells were split in four groups based on lengths, and the proportion of cells in each group is plotted. Numbers of cells analyzed were as follows: *n* = 204 for control cells (strain IM133), *n* = 256 for sgRNA-lp_1247 (strain IM141), *n* = 190 for sgRNA-lp_2217 (strain IM142), and *n* = 244 for sgRNA-lp_1247-lp_2217 (strain IM130). Plus signs (+) indicate that cells were grown in the presence of 25 ng/ml SppIP. (D) The transcription levels of genes *lp_1247* and *lp_2217* in different strains as analyzed by droplet digital PCR. In addition to *lp_2217* cells (strain IM142), *lp_1247* cells (strain IM141), and the double-knockdown cells (strain IM130), control cells with nontargeting sgRNA (strain IM133) were included in the analysis.

Using CRISPRi to knock down transcription of *acm2*, *dnaA*, and *ezrA* demonstrated the suitability of this system to phenotype key cell cycle genes. We therefore used this system to screen the phenotypes of genes which have as-yet-unknown functions in rod-shaped bacterial cells. CozE proteins have been shown to be essential for proper cell division both in oval-shaped S. pneumoniae and coccus-shaped S. aureus, but despite its conservation across the bacterial kingdom (38), their involvement in cell division has hitherto not been studied in rod-shaped bacteria. Homology searches revealed that L. plantarum WCFS1 encodes two CozE homologs; Lp_1247 (35% identity and 60% similarity to CozE from S. pneumoniae) and Lp_2217 (29% identity and 52% similarity to CozE from S. pneumoniae). We constructed CRISPRi strains targeting each of these and analyzed the morphologies of the knockdown strains by microscopy. However, no differing phenotype was observed; the cells appeared similar to wild-type cells (Fig. 6B). We then constructed a double-sgRNA vector to target both *cozE* homologs at the same time (see Materials and Methods). Note that the sgRNA vectors are designed in a way that allows multiple sgRNAs to be cloned into the vector in parallel using BglBrick cloning, thus making simultaneous knockdown of several genes possible (Fig. 6A; see also Materials and Methods). Cell length analysis of the resulting double-knockdown strain did not reveal any drastic shift in cell lengths compared to control cells, although slightly increased numbers of short (<2-µm) cells were observed (Fig. 6C).

Since we did not observe any phenotype upon depletion of the CozE homologs, we analyzed the transcriptional knockdown using ddPCR. This showed that the CRISPRi system had indeed worked as expected and that knockdown of *lp_1247* or *lp_2217* or both was achieved in the respective CRISPRi strains (Fig. 6D). From this assay, we therefore conclude that CozE homologs *lp_1247* and *lp_2217* do not seem to play any prominent role in cell division in L. plantarum.

### EloR and KhpA are necessary for proper cell elongation.

**FIG 7:**
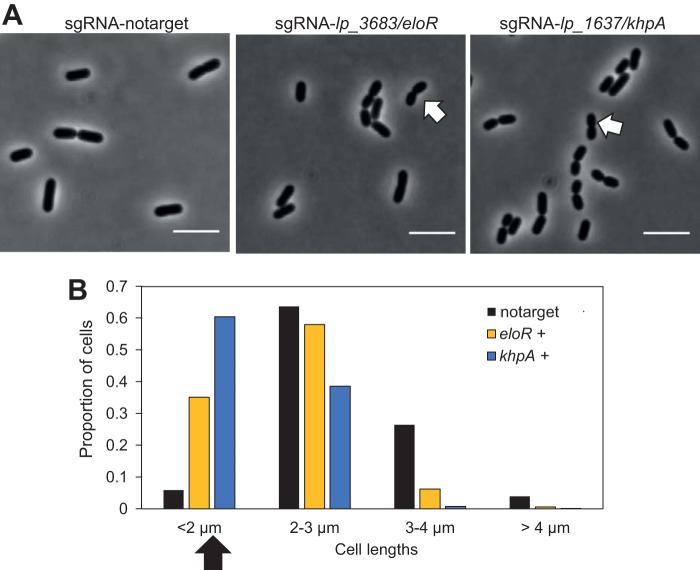
Cells depleted of *lp_3683* (*eloR* homolog) and *lp_1637* (*khpA* homolog) display reduced elongation. (A) Phase-contrast micrographs of control cells with nontargeting sgRNA (strain IM133) and of cells depleted of *lp_3683*/*eloR* (strain IM138) and *lp_1637*/*khpA* (strain IM139). Cells were grown in the presence of 25 ng/ml SppIP. Examples of short cells are indicated with white arrows. The scale bar is 5 µm. (B) Cell length analysis of corresponding strains. Cell lengths were measured using MicrobeJ (59). Cells were split in four groups based on lengths, and the proportion of cells in each group is plotted. Numbers of cells analyzed were as follows: *n* = 204 for notarget control cells (strain IM133), *n* = 689 for sgRNA-eloR cells (strain IM138), and *n* = 1025 for sgRNA-khpA cells (strain IM139). The black arrow points to the short-cell group, where cells depleted of *lp_3683*/*eloR* and *lp_1637*/*khpA* were overrepresented. Plus signs (+) indicate that cells were grown in the presence of 25 ng/ml SppIP.

EloR and KhpA are two cytoplasmic, RNA-binding proteins which have been identified in S. pneumoniae as important for proper cell elongation (39, 40, 42, 50). The two proteins form a heterocomplex, and the absence of either of them results in shorter and smaller cells. By knocking down expression of either the *eloR* homolog *lp_3683* (29% identity to the S. pneumoniae EloR protein) or the *khpA* homolog *lp_1637* (47% identity to the S. pneumoniae KhpA protein), we also observed that L. plantarum cell lengths were reduced in both cases and that the reduction was most drastic in the experiments performed with *lp_1637* (*khpA*) (Fig. 7). To our knowledge, this is the first time that these RNA-binding proteins have been investigated with respect to cell biology in rod-shaped bacteria, and the results suggest that they are important for cell elongation also in these cells. The modes of cell elongation are radically different between oval-shaped S. pneumoniae and rod-shaped cells, such as L. plantarum. While both septal cell wall synthesis and peripheral cell wall synthesis occur in the mid-cell area of ovococcal cells, elongation in rod-shaped bacteria is directed by the actin homologue MreB and occurs over most of the cell length (51). It will therefore be of great interest for future studies to unravel in detail how these proteins affect cell elongation in cells with different shapes.

### Concluding remarks.

The CRISPRi system developed in the present study is a significant addition to the genetic toolbox of L. plantarum. It allows quick and easy generation of transcriptional knockdowns, which can be used to study functions of essential genes or as part of a simple method to screen for phenotypes prior to performing time-consuming construction of knockouts or deletions. We decided to use a two-plasmid system to allow easy replacement of sgRNA sequences by inverse PCR. The design of the plasmids also allows double or multiple knockdowns to be performed simultaneously. Given the host range of the plasmids used, we also expect that the two-plasmid CRISPRi system can be directly transferred to at least two other species; L. sakei and L. curvatus.

While the CRISPRi system was shown to function well, it should also be noted that studies in E. coli have shown that CRISPRi can be toxic to the cells under certain conditions. It was shown that some sgRNA sequences were toxic and showed off-target effects when combined with high levels of dCas9 expression (52). We cannot exclude the possibility that similar issues may occur in L. plantarum, and the current CRISPRi system could be further improved with more strictly regulated promoters. Furthermore, the fact that the CRISPRi system is active even without induction could potentially result in problems in studies generating strains targeting essential genes whose reduced transcription has a detrimental effect on growth. Our results also show that the inducer concentrations needed for knockdown differ between different genes. Nevertheless, we have shown the suitability of the transcriptional knockdown system for screening for phenotypes by targeting different cell cycle genes. The initial insights into essential cell cycle processes in L. plantarum presented here form a basis for further studies of DNA replication, chromosome segregation, cell division, and cell wall synthesis in this species.

## MATERIALS AND METHODS

### Bacterial strains and growth conditions.

E. coli TOP10 or DH5α cells were grown in brain heart infusion medium (Oxoid Ltd., Carlsbad, CA) or lysogeny broth at 37°C with shaking. L. plantarum cells were grown in de Man, Rogosa, and Sharpe (MRS) broth (Oxoid) at 37°C without shaking. Solid media were prepared by addition of 1.5% (wt/vol) agar to the broth. L. lactis was used as subcloning host for plasmids containing the SH71_rep_ replicon and were transformed as previously described (53). All other plasmids were subcloned in E. coli. The final plasmids were electrotransformed into L. plantarum (54). When required, antibiotic concentrations were added as follows: for L. plantarum and L. lactis, 10 µg/ml erythromycin and 10 µg/ml chloramphenicol; for E. coli, 200 µg/ml erythromycin and 10 µg/ml chloramphenicol.

### Construction of plasmids.

To develop the CRISPR interference system, we constructed a two-plasmid system in which both plasmids are derivatives of pSIP403 (15). Plasmids were purified using a NucleoSpin plasmid miniprep kit (Macherey-Nagel GMbH & Co., Düren, Germany). All plasmids used in the study are listed in Table 1. All PCRs were performed using Q5 High-Fidelity DNA polymerase (New England Biolabs [NEB], Ipswich, MA), and the primers used for PCR amplification are listed in Table 2. Constructs were verified by DNA sequencing.

**TABLE 1:** Strains and plasmids used in this study

Strain or plasmid	Relevant characteristic(s)^*a*^	Reference or source
Strains		
E. coli TOP10	Cloning host	Thermo Fisher
E. coli DH5α	Cloning host	Laboratory stock
L. lactis Il1403	Cloning host	60
L. plantarum WCFS1	Host strain	12
L. plantarum NZ3557	WCFS1, Δ*acm2*::cat	34
L. plantarum IM167	WCFS1, pEV, pSgRNA-notarget	This study
L. plantarum IM132	WCFS1, pSIP-SH-dCas9	This study
L. plantarum IM133	WCFS1, pSIP-SH-dCas9, pSgRNA-notarget	This study
L. plantarum IM134	WCFS1, pSIP-SH-dCas9, pSgRNA-acm2	This study
L. plantarum IM137	WCFS1, pSIP-SH-dCas9, pSgRNA-dnaA	This study
L. plantarum IM188	WCFS1, pSIP-SH-dCas9, pSgRNA-ezrA	This study
L. plantarum IM141	WCFS1, pSIP-SH-dCas9, pSgRNA-lp_1247 (*cozE* homolog)	This study
L. plantarum IM142	WCFS1, pSIP-SH-dCas9, pSgRNA-lp_2217 (*cozE* homolog)	This study
L. plantarum IM130	WCFS1, pSIP-SH-dCas9, pSgRNA-lp_1247–lp_2217 (*cozE* homologs)	This study
L. plantarum IM138	WCFS1, pSIP-SH-dCas9, pSgRNA-lp_3683 (*eloR* homolog)	This study
L. plantarum IM139	WCFS1, pSIP-SH-dCas9, pSgRNA-lp_1637 (*khpA* homolog)	This study

Plasmids		
pSIP403	*spp*-based expression vector; Em^r^; 256_rep_, pUCori; P*_sppA_*::*gusA*	15
pSIP411	*spp*-based expression vector; Em^r^; SH71_rep_; P*_sppQ_*::*gusA*	16
pSIPdCas9	Em^r^; 256_rep_, pUCori; P*_sppA_*::dCas9	This study
pSIP-SH-dCas9	Em^r^; SH71_rep_; P*_sppA_*::dCas9	This study
pPEPX-P3-sgRNAluc		22
pValac	Template for chloramphenicol resistance gene (Cm^r^)	55
pEV	Em^r^; control plasmid, empty vector	61
pSgRNA-notarget	Cm^r^, 256_rep_, pUCori P3::sgRNA-notarget	This study
pSgRNA-acm2	Cm^r^ 256_rep_, pUCori P3::sgRNA-*lp_2645*	This study
pSgRNA-dnaA	Cm^r^ 256_rep_, pUCori P3::sgRNA-*lp_0001*	This study
pSgRNA-ezrA	Cm^r^ 256_rep_, pUCori P3::sgRNA-*lp_2328*	This study
pSgRNA-lp_1247	Cm^r^ 256_rep_, pUCori P3::sgRNA-*lp_1247*	This study
pSgRNA-lp_2217	Cm^r^ 256_rep_, pUCori P3::sgRNA-*lp_2217*	This study
pSgRNA-lp_1247- lp_2217	Cm^r^ 256_rep_, pUCori P3::sgRNA-*lp_1247*-*lp_2217*	This study
pSgRNA-lp_3683/eloR	Cm^r^ 256_rep_, pUCori P3::sgRNA-*lp_3683*	This study
pSgRNA-lp_1637/khpA	Cm^r^ 256_rep_, pUCori P3::sgRNA-*lp_1637*	This study

a Em, erythromycin; Cm, chloramphenicol.

**TABLE 2:** Primers used in this study

Primer and category	Sequence (5’–3’)^*a*^
Cloning	
403BamCmF	TATGCGTGCG*GGATCC*TTATTTGCTGAAAATGAGGAATTAAAAAAAGA
403SalCmR	GTGCTTTGCCGCATGC*GTCGAC*TTATAAAAGCCAGTCATTAGGCCT
SgRNA_F	GACTGGCTTTTATAAGTCGACGCATGCGGCAAAGCACTCAAAAGT
SgRNA_R	TCGAACCCGG*GGTACC*ACTTAAAAAAAAACCGCGCCCT
Cas9NcoF	AGTATGATT*CCCATGG*ATAAGAAATACTCAATAGGCTT
Cas9XhoR	TACCGAATTC*CTCGAG*GTCGACTTAGTCACCTCCT

sgRNA	
Phospho-sgRNA_promoter_R	Phosphp-5′-TATAGTTATTATACCAGGGGGACAGTGC
ezrA_lp_2328SgRNA	GTTGCCGTTCCGTCAATTGAGTTTAAGAGCTATGCTGGAAACAG
lp_p2645F	TTTCCCTTAGTCGCAGCTGCGTTTAAGAGCTATGCTGGAAACAG
mk277_sg_lp0001_dnaA	TCGAGTTGGAGTGGTTTTGCGTTTAAGAGCTATGCTGGAAACAG
mk281_sg_lp1247	ACACTTAAAAACGTGCCGATGTTTAAGAGCTATGCTGGAAACAG
mk282_sg_lp2217	CACCGTTGTTTCTGTGAATCGTTTAAGAGCTATGCTGGAAACAG
mk276_sg_lp2189_divIVA	CCAAGAACGGTTGGACTCGAGTTTAAGAGCTATGCTGGAAACAG
mk278_sg_lp3683 (eloR)	ACCGTTAGCCGTACTTGGGCGTTTAAGAGCTATGCTGGAAACAG
mk279_sg_lp1637 (spr0683)	AACTAACGGGGTGACAACCGGTTTAAGAGCTATGCTGGAAACAG

ddPCR	
Lp_1247_F	CACGATTACGAGTGTGACGA
Lp_1247_R	CTAGAAATCGTGTCGCCCAT
Lp_2217_F	CCATGGATGTTGGTCCAAGT
Lp_2217_R	CAAGATCGCATAGCCTGGAA
Lp_2645_F	ATTCTGGAAAGTGGTTGGGG
Lp_2645_R	ACTTCCGAAAAGCGTCTTGA

a Restriction sites are indicated in italics; base-pairing regions in the sgRNA primers are underlined.

### Construction of plasmid pSIP-SH-dCas9 for expression of dCas9.

To construct the plasmid containing the *dcas9* gene under the control of the inducible *sppA* promoter, the *dcas9* sequence was amplified from pLOW-dCas9 (23) using primer pair Cas9NcoF/Cas9XhoR. The amplicon was cloned into NcoI/XhoI-digested pSIP403 (15) using an In-Fusion HD cloning kit (Clonetech Laboratories, Mountain View, CA), resulting in plasmid pSIP-dCas9. Then, to exchange the 256_rep_ replicon with the SH71_rep_ replicon, the SH71_rep_ replicon was excised from plasmid pSIP411 (16) using BamHI and XhoI and was ligated into the same sites of pSIP-dCas9, resulting in plasmid pSIP-SH-dCas9.

### Construction of plasmids for expression of sgRNAs.

We initially constructed a plasmid containing sgRNA-notarget under the control of a constitutive promoter. The sgRNA-notarget cassette was amplified from plasmid pPEPX-P3-sgRNAluc (22) with primer pair SgRNA_F/SgRNA_R, and the chloramphenicol resistance gene with the cognate promoter was amplified from plasmid pValac (55) using primer pair 403BamCmF/403SalCmR. The two fragments (with 36 overlapping base pairs) were fused by PCR, using the outer primers SgRNA_F and 403SalCmR. The 1.4-kb amplicon was subsequently cloned into BamHI/Acc65I-digested pSIP403 (15), using an In-Fusion HD cloning kit (Clontech Laboratories, Mountain View, CA), resulting in plasmid pSgRNA-notarget. This plasmid was used as a starting point for the insertion of gene-specific sgRNAs.

Gene-specific base-pairing regions for the sgRNAs were selected according to criteria previously used for other bacteria (22, 56). Shortly, we used CRISPR Primer Designer (57) to find potential protospacer-adjacent motif (PAM) sites (5′-NGG-3′) and adjacent base-pairing regions close to the 5′ end of the gene of interest. Base-pairing sequences binding to the nontemplate DNA strand were selected. BLAST searches (using the PAM-proximal 12 bp of the sgRNA as the query) against the WCFS1 genome were performed to ensure that there were no secondary target site on the genome. To verify that the base-pairing region does not interfere with the secondary structure of the dCas9 handle region, RNAfold from the ViennaRNA package was used to predict the sgRNA secondary structure.

New sgRNA plasmids were then constructed using inverse PCR. The base-pairing regions were introduced as overhangs in the forward primer, while the reverse primer was 5′-phosphorylated. Following inverse PCR, the template plasmid was digested using DpnI at 37°C for 2 h. The amplified PCR fragment were self-ligated using T4 DNA ligase (NEB) following the manufacture’s protocol and transformed into E. coli. Purified sgRNA plasmids were verified by sequencing and transformed into electrocompetent L. plantarum harboring pSIP-SH-dCas9.

### Construction of double-sgRNA plasmid.

A plasmid for simultaneous depletion of *lp_1247* and *lp_2217* (pSgRNA-lp_1247-lp_2217) was made using BglBrick cloning (58). Plasmid pSgRNA-lp_1247 was digested using EcoRI and BglII, while the sgRNA-lp_2217 fragment was digested from plasmid pSgRNA-lp_2217 using EcoRI and BamHI and was ligated into the EcoRI/BglII sites of plasmid pSgRNA-lp_1247 to generate the double-sgRNA plasmid (see also Fig. 6A).

### Total RNA extraction and cDNA synthesis.

Overnight cultures of L. plantarum harboring pSIP-SH-dCas9 and sgRNA plasmids were diluted in fresh MRS medium with appropriate antibiotics to an OD_600_ of 0.01 and induced with 25 ng/ml IP-67 (SppIP) (45) (Caslo, Lyngby, Denmark). In dose-response experiments, the inducer concentration range from 0 to 25 ng/ml. When cultures reached an OD_600_ of 0.4, 5 ml of culture was added to an equal volume of Bacteria Protect (Qiagen, Hilden, Germany) and the culture was subsequently harvested by centrifugation for 10 min at 5000 × *g* and 22°C. Total RNA was extracted using an RNeasy minikit (Qiagen). Cell pellets were resuspended in 700 µl RLT buffer (Qiagen) with 1.5% β-mercaptoethanol. For lysis by mechanical disruption, the suspensions were transferred to 2 ml lysing matrix B tubes (MP Biomedicals) and placed in a FastPrep-24 instrument (MP Biomedicals) at 6.5 m/s for 30 s. The agitation was repeated three times with a 1-min pause between agitations. Further steps were carried out according to the protocol provided by the manufacturer (Qiagen). After extraction, a Heat&Run genomic DNA (gDNA) removal kit (ArcticZymes, Tromsø, Norway) was used according to the manufacturer’s instructions to remove residual gDNA. The concentrations of the RNA samples were determined using a NanoDrop spectrophotometer (Thermo Fisher Scientific Inc, Waltham, MA). cDNA was synthetized by the use of iScript Reverse Transcription Supermix (Bio-Rad, Hercules, CA, USA), where 100 ng RNA was used as the template. Negative controls (not containing reverse transcriptase) were prepared for each sample to check that all gDNA had been removed. Reactions were set up as described by the manufacturer. To prevent saturation of template in droplet digital PCR reactions, all cDNA samples were diluted 100×.

### Droplet digital PCR (ddPCR).

ddPCR was performed by mixing 11 µl of 2× EvaGreen Supermix (Bio-Rad), 1 µl of 2 µM stocks for each primer (see Table 2), and distilled water (dH_2_O) to reach a total volume of 20 µl for each reaction. The mix was dispersed into PCR strips, and then 2 µl of the template was added. The reaction mixtures were loaded into a QX200 system (Bio-Rad) for droplet generation. Droplet generation required 20 µl of sample and 70 µl of droplet generation oil for EvaGreen (Bio-Rad). Droplets were created in a volume of 40 µl and were transferred to a High-Profile 96-well PCR plate (Bio-Rad). The plate was sealed using a PX1 PCR plate sealer (Bio-Rad) and put into a thermocycler with a ramp rate of 2°C/s. The cycling program was 95°C for 10 min, 40 cycles of 95°C for 30 s and 60°C for 1 min, and then 4°C for 5 min and 90°C for 5 min, followed by an optional infinite hold at 4°C. After amplification, droplet signals were analyzed using a QX200 reader (Bio-Rad). Data were analyzed using QuantaSoft Analysis Pro with the default setup. Only results from wells where RNA samples were partitioned in >10,000 droplets were included in further calculations.

### Phase-contrast and fluorescence microscopy analysis.

To prepare samples for microcopy analysis, overnight cultures of L. plantarum were diluted in fresh MRS medium with appropriate antibiotics to an OD_600_ of 0.01 and induced with 25 ng/ml SppIP (Caslo). When the OD_600_ reached 0.4, 80-µl samples were collected and subsequently mixed with 31.25 µg/ml DAPI for staining of DNA. A Zeiss AxioObserver and ZEN blue software were used for microscopy, and images were taken with an Orca-Flash4.0 V2 Digital complementary metal-oxide semiconductor (CMOS) camera (Hamamatsu Photonics) through a 100× PC objective. An HPX 120 Illuminator was used as a light source for fluorescence microscopy analysis.

### Growth assays.

Growth assays were conducted in a Synergy H1 hybrid reader (BioTek). Overnight cultures of L. plantarum were diluted to an OD_600_ of 0.01 in fresh MRS medium containing appropriate antibiotics. Cell suspensions were dispersed in a 96-well microtiter plate with 270 µl culture in each well. Cultures were induced with 25 ng/ml SppIP (Caslo). OD_600_ was measured every 10 min at 37°C during growth.
